# Supplementary material for: Inferring network properties from time series using transfer entropy and mutual information: Validation of multivariate versus bivariate approaches
Source: Netw Neurosci. 2021 Apr 27;5(2):373–404. doi: 10.1162/netn_a_00178 (PMC8233116; doi:10.1162/netn_a_00178)
Supplement: Supplementary file 1 [file netn-05-373-s001.pdf]

Novelli, L., Lizier, J. T. (2020). Supporting information for "Inferring network properties from time series using transfer entropy and mutual information: validation of multivariate versus bivariate approaches." *Network Neuroscience.* Advance publication. https://doi.org/10.1162/netn_a_00178

SUPPORTING INFORMATION

Local and global efficiency

The crucial limitation of shortest path length measures (and of the derived small-world coefficient) is being only defined for connected networks. Therefore, the analogous global efficiency measure (Latora & Marchiori, 2001) is often used to overcome this shortcoming (also see (Zanin, 2015) for an alternative measure of small-worldness based on global efficiency). The related local efficiency measure can instead be regarded as analogous to the clustering coefficient (Latora & Marchiori, 2001). Complementing the results in the main text, we report the global and local efficiency of small-world networks (Figure 16), scale-free networks (Figure 17), and two real macaque connectomes (Figure 18 and Figure 19).

Clustering coefficient of scale-free networks

Plotting the clustering coefficient values of individual nodes instead of the average shows that the low clustering values are consistently overestimated by bivariate methods (which is the most prominent effect affecting the average), while high clustering values are underestimated (Figure 20).

Rich-club coefficient and assortativity of scale-free networks

The rich-club coefficient measures the extent to which high-degree nodes connect to each other (Colizza et al., 2006). Instead of choosing a specific threshold to define high-degree nodes, the rich-club coefficient is plotted in Figure 21 for a range of thresholds (non-normalised values). The rich-club coefficient is overestimated by bivariate MI and TE across all thresholds, although the effect is less prominent than on other network properties.

Assortativity (or assortative mixing) is a preference for nodes to attach to others with similar degree. The in-degree assortativity coefficient is the Pearson correlation coefficient of degree between pairs of linked nodes. Positive values indicate a correlation between nodes of similar in-degree, while negative values indicate relationships between nodes of different in-degree. As shown in Figure 22, the scale-free networks obtained via preferential attachment are disassortative (i.e., they have negative assortativity coefficients). Bivariate and multivariate TE accurately reproduce the assortativity of the real networks (ground truth), while bivariate MI consistently underestimate it.

Reducing α and sample ROC curve

Reducing the critical statistical significance level α clearly helps to reduce false positives, for any approach. However, what distinguishes the multivariate approach on this point is two-fold.

First, the significance level α has direct meaning regarding the false-positive rate (FPR) under idealised conditions, implying that a well-calibrated test should produce a FPR consistent with α. This is the case for multivariate TE under the ideal conditions investigated here, but not for bivariate measures, where the FPR is inflated drastically above the requested level. Certainly one can decrease α, but the experimenter has no a-priori insights regarding what to set it to.

Second, even though one can in principle decrease the FPR for bivariate measures by decreasing the significance level, a very large number of true positives would also be missed

by doing so, and therefore the desired reflection of the relationships in the underlying structure would not be achieved.

We can compare the receiver operating characteristic (ROC) curve for bivariate measures to the multivariate TE for the experiment on a neural mass model, shown in Figure 23. Note that for multivariate TE, each point on the scatter plot is generated from separate runs with different α parameters rather than a single run (since the recall and FPR are functions of the whole inferred network). We see that the FPRs in this experiment are substantially inflated over the experiments under idealised conditions, as discussed in the main text. Crucially though, whilst these are inflated for all measures, the contrasts in FPRs between the approaches are quite large when converted to the numbers of spurious links inferred. This can be seen visually in Figure 15. The multivariate TE operates at a much larger true-positive rate than the bivariate measures for the same FPR; therefore, simply reducing α for bivariate measures is not going to result in as effective a performance as multivariate TE.

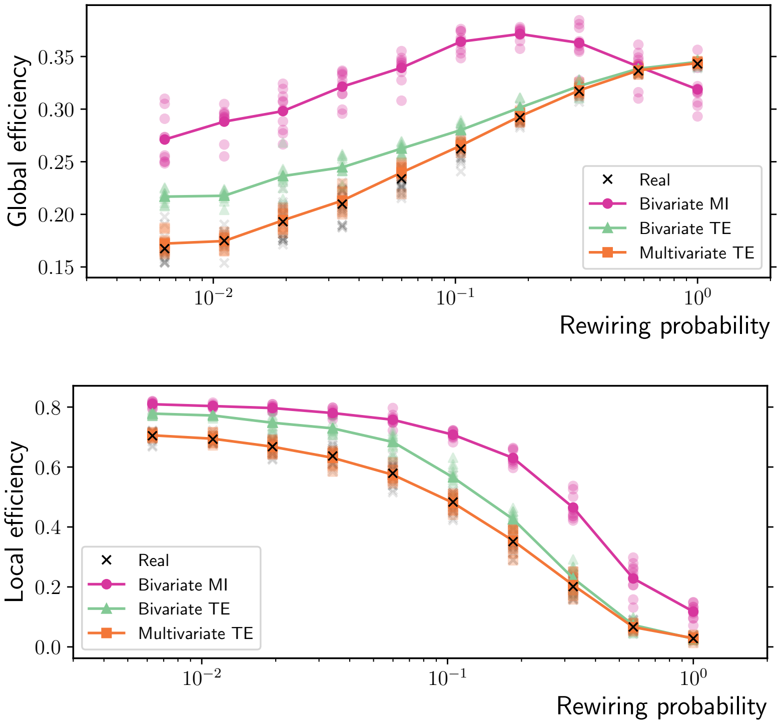

Figure 16. Global and local efficiency as a function of the rewiring probability in Watts-Strogatz ring networks (N=100 nodes and T=10 000 time samples). Multivariate TE reconstructs networks having the same efficiency as the real topologies (ground truth). On the other hand, bivariate MI and TE produce significant overestimates due to spurious links. These create shortcuts across the network (inflating the global efficiency in the top panel) and form spurious triangular cliques (inflating the local efficiency in the bottom panel), particularly on lattice-like topologies (low rewiring probability). For each value of the rewiring probability, the results for 10 simulations on different network realisations are presented (low-opacity markers) in addition to the mean values (solid markers).

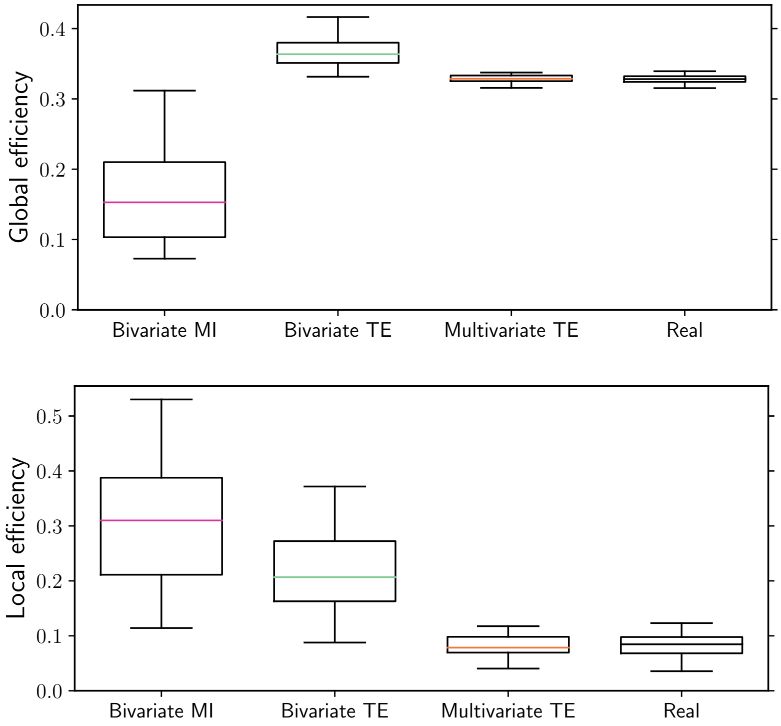

Figure 17. Global and local efficiency in scale-free networks obtained via preferential attachment (N=200 nodes and T=10 000 time samples). Multivariate TE is the only algorithm able to preserve the efficiency of the real networks (ground truth), while bivariate TE and MI consistently overestimate it. The box-whiskers plots summarise the results over 10 simulations, with median values indicated in colour.

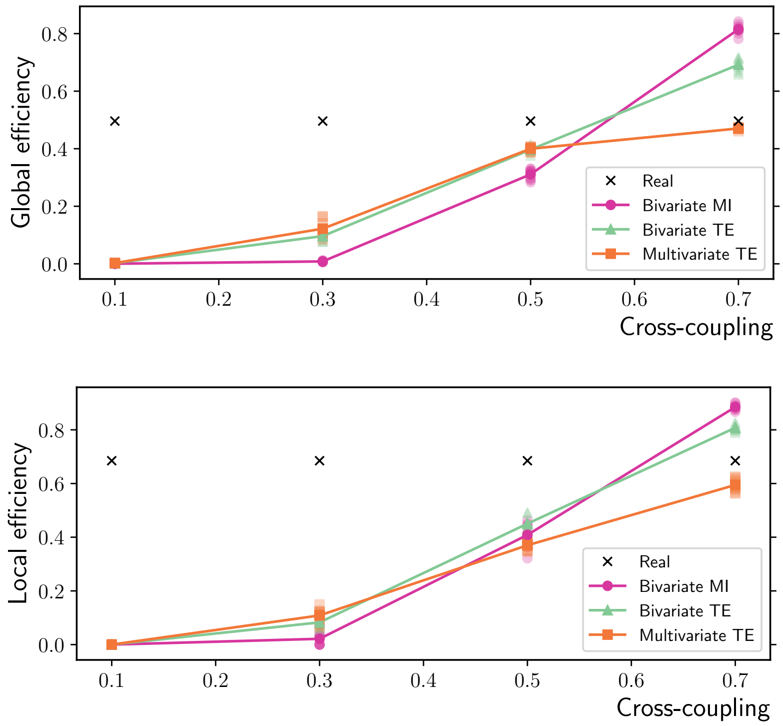

Figure 18. Global and local efficiency as a function of coupling weight in a real macacque connectome (N=71 nodes and T=10 000 time samples). All inference algorithms produce underestimates for low coupling. For stronger coupling, multivariate TE converges to the real global and local efficiency of the underlying networks (ground truth), while bivariate methods overestimate both measures. For each value of the cross-coupling weights, the results for 10 simulations from random initial conditions are presented (low-opacity markers) in addition to the mean values (solid markers).

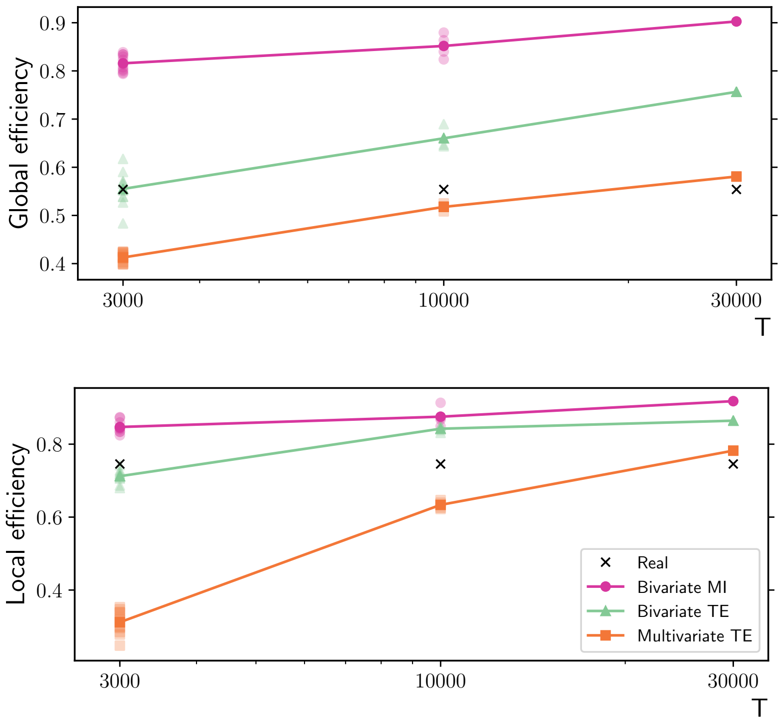

Figure 19. Global and local efficiency as a function of number of time samples T in a real macacque connectome with N=76 nodes. Bivariate MI consistently overestimates both measures. Bivariate TE produce the most accurate estimates for shorter time series but tends to overestimate both efficiency measures as more data is provided. Multivariate TE significantly underestimates both measures for shorter time series, but is the only method that approximately converges to real values as more data is provided. The results for all simulations are presented (low-opacity markers) in addition to the mean values (solid markers).

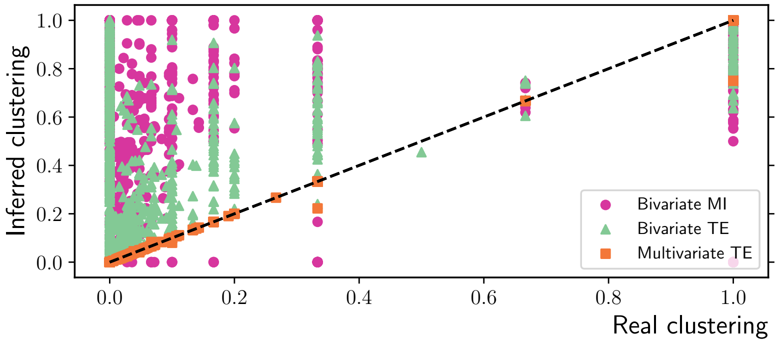

Figure 20. Inferred vs. real clustering coefficient of individual nodes in scale-free networks obtained via preferential attachment (N=200 nodes and T=10 000 time samples). Multivariate TE accurately reproduces the clustering coefficient of the real networks (ground truth), while bivariate methods overestimate low clustering values and underestimate high clustering values. The results are collected over 10 simulations on different network realisations.

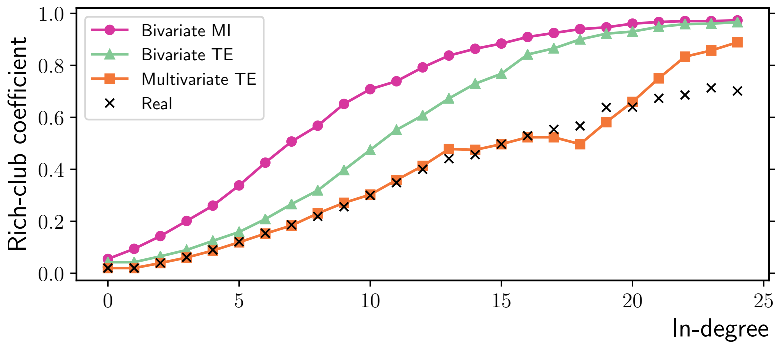

Figure 21. Rich-club coefficient in scale-free networks obtained via preferential attachment (N=200 nodes and T=10 000 time samples). Multivariate TE accurately reproduces the rich-club coefficient of the real networks (ground truth) for lower in-degree thresholds, while bivariate TE and MI consistently overestimate it. Mean values over 10 simulations.

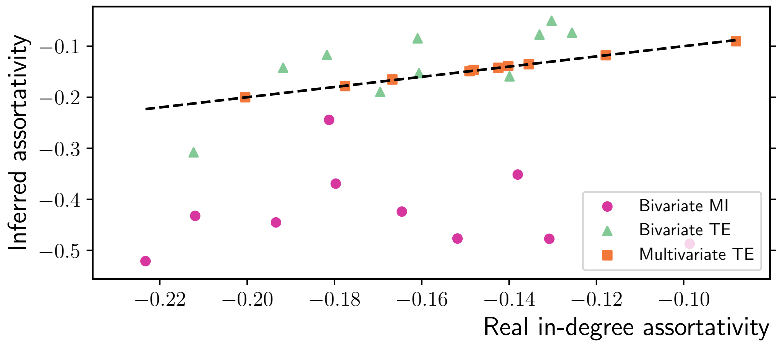

Figure 22. In-degree assortativity coefficient in scale-free networks obtained via preferential attachment (N=200 nodes and T=10 000 time samples). Bivariate and multivariate TE accurately reproduce the assortativity of the real networks (ground truth), while bivariate MI consistently underestimate it. The black dashed line represents the identity between real and inferred values. The results are shown for 10 different network realisations.

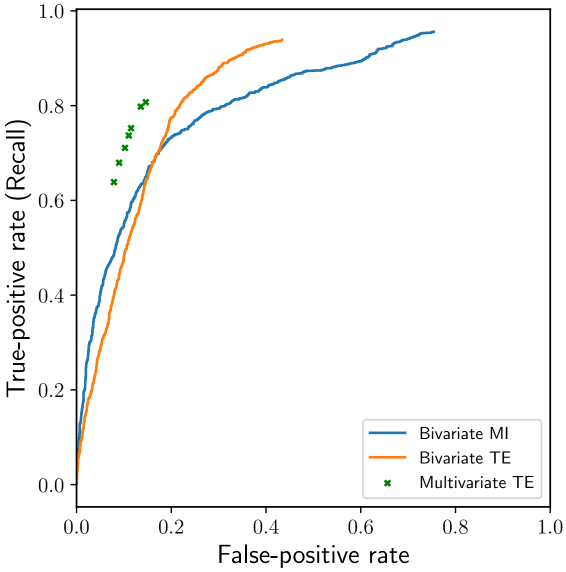

Figure 23. Partial receiver operating characteristic curve. For multivariate TE, note that the whole algorithm must be re-run for each α, and so a limited locus of the ROC curve is plotted for it. The specific threshold values tested for multivariate TE are $\alpha \in \{0.00001, 0.0001, 0.001, 0.005, 0.01, 0.05, 0.1\}$.

REFERENCES

Aertsen, A. M., Gerstein, G. L., Habib, M. K., & Palm, G. (1989). Dynamics of neuronal firing correlation: modulation of ”effective connectivity”. *Journal of Neurophysiology*, *61*(5), 900–917. doi: 10.1152/jn.1989.61.5.900

Alstott, J., Bullmore, E., & Plenz, D. (2014). powerlaw: A Python Package for Analysis of Heavy-Tailed Distributions. *PLoS ONE*, *9*(1), e85777. doi: 10.1371/journal.pone.0085777

Aquino, K. M., Fulcher, B. D., Parkes, L., Sabaroedin, K., & Fornito, A. (2020). Identifying and removing widespread signal deflections from fMRI data: Rethinking the global signal regression problem. *NeuroImage*, *212*, 116614. doi: 10.1016/j.neuroimage.2020.116614

Atay, F. M., & Karabacak, Ö. (2006). Stability of Coupled Map Networks with Delays. *SIAM Journal on Applied Dynamical Systems*, *5*(3), 508–527. doi: 10.1137/060652531

Barabási, A.-L., & Albert, R. (1999). Emergence of Scaling in Random Networks. *Science*, *286*(5439), 509–512. doi: 10.1126/science.286.5439.509

Barnett, L., Barrett, A. B., & Seth, A. K. (2009). Granger Causality and Transfer Entropy Are Equivalent for Gaussian Variables. *Physical Review Letters*, *103*(23), 238701. doi: 10.1103/PhysRevLett.103.238701

Barnett, L., & Seth, A. K. (2017). Detectability of Granger causality for subsampled continuous-time neurophysiological processes. *Journal of Neuroscience Methods*, *275*, 93–121. doi: 10.1016/j.jneumeth.2016.10.016

Bassett, D. S., & Sporns, O. (2017). Network neuroscience. *Nature Neuroscience*, *20*(3), 353–364. doi: 10.1038/nn.4502

Bettinardi, R. G., Deco, G., Karlaftis, V. M., Van Hartevelt, T. J., Fernandes, H. M., Kourtzi, Z., … Zamora-López, G. (2017). How structure sculpts function: Unveiling the contribution of anatomical connectivity to the brain's spontaneous correlation structure. *Chaos: An Interdisciplinary Journal of Nonlinear Science*, *27*(4), 047409. doi: 10.1063/1.4980099

Bialonski, S., Horstmann, M.-T., & Lehnertz, K. (2010). From brain to earth and climate systems: Small-world interaction networks or not? *Chaos: An Interdisciplinary Journal of Nonlinear Science*, *20*(1), 013134. doi: 10.1063/1.3360561

Blondel, V. D., Guillaume, J.-L., Lambiotte, R., & Lefebvre, E. (2008). Fast unfolding of communities in large networks. *Journal of Statistical Mechanics: Theory and Experiment*, *2008*(10), P10008. doi: 10.1088/1742-5468/2008/10/P10008

Bossomaier, T., Barnett, L., Harré, M., & Lizier, J. T. (2016). *An Introduction to Transfer Entropy*. Cham: Springer International Publishing. doi: 10.1007/978-3-319-43222-9

Budden, D. M., & Crampin, E. J. (2016). Information theoretic approaches for inference of biological networks from continuous-valued data. *BMC Systems Biology*, *10*(1), 89. doi: 10.1186/s12918-016-0331-y

Cliff, O. M., Novelli, L., Fulcher, B. D., Shine, J. M., & Lizier, J. T. (2020). *Exact Inference of Linear Dependence Between Multiple Autocorrelated Time Series.* Retrieved from `http://arxiv.org/abs/2003.03887`

Colizza, V., Flammini, A., Serrano, M. A., & Vespignani, A. (2006). Detecting rich-club ordering in complex networks. *Nature Physics*, *2*(2), 110–115. doi: 10.1038/nphys209

Cover, T. M., & Thomas, J. A. (2005). *Elements of Information Theory* (2nd ed.). Hoboken, NJ, USA: John Wiley & Sons, Inc. doi: 10.1002/047174882X

Faes, L., Nollo, G., & Porta, A. (2011). Information-based detection of nonlinear Granger causality in multivariate processes via a nonuniform embedding technique. *Physical Review E*, *83*(5), 051112. doi: 10.1103/PhysRevE.83.051112

Fagiolo, G. (2007). Clustering in complex directed networks. *Physical Review E*, *76*(2), 026107. doi: 10.1103/PhysRevE.76.026107

FitzHugh, R. (1961). Impulses and physiological states in theoretical models of nerve membrane [Journal Article]. *Biophysical Journal*, *1*(6), 445-466.

Fornito, A., Zalesky, A., & Bullmore, E. T. (2016). *Fundamentals of Brain Network Analysis* (1st ed.). San Diego: Academic Press. doi: 10.1016/B978-0-12-407908-3.09999-4

Gillis, J., & Pavlidis, P. (2011). The role of indirect connections in gene networks in predicting function. *Bioinformatics*, *27*(13), 1860–1866. doi: 10.1093/bioinformatics/btr288

Goni, J., van den Heuvel, M. P., Avena-Koenigsberger, A., Velez de Mendizabal, N., Betzel, R. F., Griffa, A., … Sporns, O. (2014). Resting-brain functional connectivity predicted by analytic measures of network communication. *Proceedings of the National Academy of Sciences*, *111*(2), 833–838. doi: 10.1073/pnas.1315529111

Granger, C. W. J. (1969). Investigating Causal Relations by Econometric Models and Cross-spectral Methods. *Econometrica*, *37*(3), 424–438. doi: 10.2307/1912791

He, Y., Chen, Z. J., & Evans, A. C. (2007). Small-World Anatomical Networks in the Human Brain Revealed by Cortical Thickness from MRI. *Cerebral Cortex*, *17*(10), 2407–2419. doi: 10.1093/cercor/bhl149

Hilgetag, C. C., & Goulas, A. (2015). Is the brain really a small-world network? *Brain Structure and Function*, *221*(4), 2361–2366. doi: 10.1007/s00429-015-1035-6

Hlinka, J., Hartman, D., & Paluš, M. (2012). Small-world topology of functional connectivity in randomly connected dynamical systems. *Chaos: An Interdisciplinary Journal of Nonlinear Science*, *22*(3), 033107. doi: 10.1063/1.4732541

Honey, C. J., Kotter, R., Breakspear, M., & Sporns, O. (2007). Network structure of cerebral cortex shapes functional connectivity on multiple time scales. *Proceedings of the National Academy of Sciences*, *104*(24), 10240–10245. doi: 10.1073/pnas.0701519104

Humphries, M. D., & Gurney, K. (2008). Network 'Small-World-Ness': A Quantitative Method for Determining Canonical Network Equivalence. *PLoS ONE*, *3*(4), e0002051. doi: 10.1371/journal.pone.0002051

Kim, P., Rogers, J., Sun, J., & Bollt, E. M. (2016). Causation Entropy Identifies Sparsity Structure for Parameter Estimation of Dynamic Systems. *Journal of Computational and Nonlinear Dynamics*, *12*(1), 011008. doi: 10.1115/1.4034126

Kötter, R. (2004). Online Retrieval, Processing, and Visualization of Primate Connectivity Data From the CoCoMac Database. *Neuroinformatics*, *2*(2), 127–144. doi: 10.1385/NI:2:2:127

Kugiumtzis, D. (2013). Direct-coupling information measure from nonuniform embedding. *Physical Review E*, *87*(6), 062918. doi: 10.1103/PhysRevE.87.062918

Langford, E., Schwertman, N., & Owens, M. (2001). Is the Property of Being Positively Correlated Transitive? *The American Statistician*, *55*(4), 322–325. doi: 10.1198/000313001753272286

Latora, V., & Marchiori, M. (2001). Efficient Behavior of Small-World Networks. *Physical Review Letters*, *87*(19), 198701. doi: 10.1103/PhysRevLett.87.198701

Li, M., Han, Y., Aburn, M. J., Breakspear, M., Poldrack, R. A., Shine, J. M., & Lizier, J. T. (2019). Transitions in information processing dynamics at the whole-brain network level are driven by alterations in neural gain. *PLOS Computational Biology*, *15*(10), e1006957. doi: 10.1371/journal.pcbi.1006957

Lizier, J. T. (2014). JIDT: An Information-Theoretic Toolkit for Studying the Dynamics of Complex Systems. *Frontiers in Robotics and AI*, *1*, 11. doi: 10.3389/frobt.2014.00011

Lizier, J. T., Heinzle, J., Horstmann, A., Haynes, J.-D., & Prokopenko, M. (2011). Multivariate information-theoretic measures reveal directed information structure and task relevant changes in fMRI connectivity. *Journal of Computational Neuroscience*, *30*(1), 85–107. doi: 10.1007/s10827-010-0271-2

Lizier, J. T., & Rubinov, M. (2012). Multivariate construction of effective computational networks from observational data. *Max Planck Institute: Preprint*.

Maier, B. F. (2019). Generalization of the small-world effect on a model approaching the Erdős–Rényi random graph. *Scientific Reports*, *9*(1), 9268. doi: 10.1038/s41598-019-45576-3

Marinazzo, D., Wu, G., Pellicoro, M., Angelini, L., & Stramaglia, S. (2012). Information Flow in Networks and the Law of Diminishing Marginal Returns: Evidence from Modeling and Human Electroencephalographic Recordings. *PLoS ONE*, *7*(9), e45026. doi: 10.1371/journal.pone.0045026

Montalto, A., Faes, L., & Marinazzo, D. (2014). MuTE: A MATLAB Toolbox to Compare Established and Novel Estimators of the Multivariate Transfer Entropy. *PLoS ONE*, *9*(10), e109462. doi: 10.1371/journal.pone.0109462

Neal, Z. P. (2017). How small is it? Comparing indices of small worldliness. *Network Science*, *5*(1), 30–44. doi: 10.1017/nws.2017.5

Newman, M. E. J., & Girvan, M. (2004). Finding and evaluating community structure in networks. *Physical Review E*, *69*(2), 026113. doi: 10.1103/PhysRevE.69.026113

Novelli, L., Atay, F. M., Jost, J., & Lizier, J. T. (2020). Deriving pairwise transfer entropy from network structure and motifs. *Proceedings of the Royal Society A: Mathematical, Physical and Engineering Sciences*, *476*(2236), 20190779. doi: 10.1098/rspa.2019.0779

Novelli, L., Wollstadt, P., Mediano, P., Wibral, M., & Lizier, J. T. (2019). Large-scale directed network inference with multivariate transfer entropy and hierarchical statistical testing. *Network Neuroscience*, *3*(3), 827–847. doi: 10.1162/netn_a_00092

Orlandi, J. G., Stetter, O., Soriano, J., Geisel, T., & Battaglia, D. (2014). Transfer Entropy Reconstruction and Labeling of Neuronal Connections from Simulated Calcium Imaging. *PLoS ONE*, *9*(6), e98842. doi: 10.1371/journal.pone.0098842

Papo, D., Zanin, M., Martínez, J. H., & Buldú, J. M. (2016). Beware of the Small-World Neuroscientist! *Frontiers in Human Neuroscience*, *10*(March), 1–4. doi: 10.3389/fnhum.2016.00096

Pernice, V., Staude, B., Cardanobile, S., & Rotter, S. (2011). How Structure Determines Correlations in Neuronal Networks. *PLOS Computational Biology*, *7*(5), e1002059. doi: 10.1371/journal.pcbi.1002059

Razi, A., Kahan, J., Rees, G., & Friston, K. J. (2015). Construct validation of a DCM for resting state fMRI. *NeuroImage*, *106*, 1–14. doi: 10.1016/j.neuroimage.2014.11.027

Rubinov, M., & Sporns, O. (2010). Complex network measures of brain connectivity: Uses and interpretations. *NeuroImage*, *52*(3), 1059–1069. doi: 10.1016/j.neuroimage.2009.10.003

Runge, J. (2018). Causal network reconstruction from time series: From theoretical assumptions to practical estimation. *Chaos*, *28*(7), 075310. doi: 10.1063/1.5025050

Runge, J., Nowack, P., Kretschmer, M., Flaxman, S., & Sejdinovic, D. (2018). *Detecting causal associations in large nonlinear time series datasets.*

Sanz Leon, P., Knock, S. A., Woodman, M. M., Domide, L., Mersmann, J., McIntosh, A. R., & Jirsa, V. (2013). The Virtual Brain: a simulator of primate brain network dynamics. *Frontiers in Neuroinformatics*, *7*(MAY). doi: 10.3389/fninf.2013.00010

Schreiber, T. (2000). Measuring Information Transfer. *Physical Review Letters*, *85*(2), 461–464. doi: 10.1103/PhysRevLett.85.461

Schwarze, A. C., & Porter, M. A. (2020). Motifs for processes on networks.

Shannon, C. E. (1948). A Mathematical Theory of Communication. *Bell System Technical Journal*, *27*(3), 379–423. doi: 10.1002/j.1538-7305.1948.tb01338.x

Shine, J. M. (2018). *Gain_topology.* `https://github.com/macshine/gain_topology`.

Shine, J. M., Aburn, M. J., Breakspear, M., & Poldrack, R. A. (2018). The modulation of neural gain facilitates a transition between functional segregation and integration in the brain. *eLife*, *7*, 1–16. doi: 10.7554/eLife.31130

Stetter, O., Battaglia, D., Soriano, J., & Geisel, T. (2012). Model-Free Reconstruction of Excitatory Neuronal Connectivity from Calcium Imaging Signals. *PLoS Computational Biology*, *8*(8), e1002653. doi: 10.1371/journal.pcbi.1002653

Stramaglia, S., Cortes, J. M., & Marinazzo, D. (2014). Synergy and redundancy in the Granger causal analysis of dynamical networks. *New Journal of Physics*, *16*(10), 105003. doi: 10.1088/1367-2630/16/10/105003

Sun, J., Taylor, D., & Bollt, E. M. (2015). Causal Network Inference by Optimal Causation Entropy. *SIAM Journal on Applied Dynamical Systems*, *14*(1), 73–106. doi: 10.1137/140956166

Takens, F. (1981). Detecting strange attractors in turbulence. In D. Rand & L. Young (Eds.), *Dynamical systems and turbulence* (pp. 366–381). Springer Berlin Heidelberg. doi: 10.1007/BFb0091924

Telesford, Q. K., Joyce, K. E., Hayasaka, S., Burdette, J. H., & Laurienti, P. J. (2011). The ubiquity of small-world networks. *Brain Connectivity*, *1*(5), 367-375. doi: 10.1089/brain.2011.0038

van den Heuvel, M., Stam, C., Boersma, M., & Hulshoff Pol, H. (2008). Small-world and scale-free organization of voxel-based resting-state functional connectivity in the human brain. *NeuroImage*, *43*(3), 528–539. doi: 10.1016/j.neuroimage.2008.08.010

Virkar, Y., & Clauset, A. (2014). Power-law distributions in binned empirical data. *The Annals of Applied Statistics*, *8*(1), 89–119. doi: 10.1214/13-AOAS710

Vlachos, I., & Kugiumtzis, D. (2010). Nonuniform state-space reconstruction and coupling detection. *Physical Review E*, *82*(1), 016207. doi: 10.1103/PhysRevE.82.016207

Watts, D. J., & Strogatz, S. H. (1998). Collective dynamics of 'small-world' networks. *Nature*, *393*(6684), 440–442. doi: 10.1038/30918

Wibral, M., Rahm, B., Rieder, M., Lindner, M., Vicente, R., & Kaiser, J. (2011). Transfer entropy in magnetoencephalographic data: Quantifying information flow in cortical and cerebellar networks. *Progress in Biophysics and Molecular Biology*, *105*(1-2), 80–97. doi: 10.1016/j.pbiomolbio.2010.11.006

Wibral, M., Vicente, R., & Lizier, J. T. (2014). *Directed Information Measures in Neuroscience*. Berlin, Heidelberg: Springer Berlin Heidelberg. doi: 10.1007/978-3-642-54474-3

Wollstadt, P., Lizier, J. T., Vicente, R., Finn, C., Martínez-Zarzuela, M., Mediano, P., . . . Wibral, M. (2019). IDTxl: The Information Dynamics Toolkit xl: a Python package for the efficient analysis of multivariate information dynamics in networks. *Journal of Open Source Software*, *4*(34), 1081.

Xia, C. H., Ma, Z., Cui, Z., Bzdok, D., Thirion, B., Bassett, D. S., . . . Witten, D. M. (2020). Multi-scale network regression for brain-phenotype associations. *Human Brain Mapping*, 1–14. doi: 10.1002/hbm.24982

Young, M. P. (1993). The organization of neural systems in the primate cerebral cortex. *Proceedings of the Royal Society of London. Series B: Biological Sciences*, *252*(1333), 13–18. doi: 10.1098/rspb.1993.0040

Zalesky, A., Fornito, A., & Bullmore, E. (2012). On the use of correlation as a measure of network connectivity. *NeuroImage*, *60*(4), 2096–2106. doi: 10.1016/j.neuroimage.2012.02.001

Zalesky, A., Fornito, A., Cocchi, L., Gollo, L. L., van den Heuvel, M. P., & Breakspear, M. (2016). Connectome sensitivity or specificity: which is more important? *NeuroImage*, *142*, 407–420. doi: 10.1016/j.neuroimage.2016.06.035

Zalesky, A., Fornito, A., Harding, I. H., Cocchi, L., Yücel, M., Pantelis, C., & Bullmore, E. T. (2010). Whole-brain anatomical networks: Does the choice of nodes matter? *NeuroImage*, *50*(3), 970–983. doi: 10.1016/j.neuroimage.2009.12.027

Zanin, M. (2015). On alternative formulations of the small-world metric in complex networks.

Zhou, S., & Mondragon, R. (2004). The Rich-Club Phenomenon in the Internet Topology. *IEEE Communications Letters*, *8*(3), 180–182. doi: 10.1109/LCOMM.2004.823426
